# The tracking of active travel and its relationship with body composition in UK adolescents

**DOI:** 10.1016/j.jth.2015.09.005

**Published:** 2015-12

**Authors:** Catherine L. Falconer, Sam D. Leary, Angie S. Page, Ashley R Cooper

**Affiliations:** a National Institute for Health Research, Bristol Biomedical Research Unit in Nutrition, Diet and Lifestyle, Level 3 University Hospitals Bristol Education and Research Centre, Upper Maudlin Street, BS2 8AE, Bristol, UK; b Centre for Exercise, Nutrition and Health Sciences, School for Policy Studies, 8 Priory Road, University of Bristol, BS8 1TZ, Bristol, UK

**Keywords:** Active travel, Body composition, Walking, Cycling, School, Adolescence

## Abstract

**Background:**

To examine the tracking of active travel through adolescence, and its association with body mass index (BMI) and fat mass at age 17 in a UK cohort.

**Methods:**

We analysed data collected from the Avon Longitudinal Study of Parents and Children (ALSPAC). The analyses include all participants with self-reported travel mode to school at ages 12, 14 and 16 years, and measured height, weight and body composition at age 17 (*n*=2,026). Tracking coefficients were calculated for individual travel behaviours (including walking and cycling) through adolescence using Generalised Estimating Equations. Linear regression analyses examined associations between travel pattern (consistently passive, consistently active, active at two time points or active at one time point), BMI, and DXA-measured fat mass (expressed as internally derived standard deviation scores) at 17 years. Analyses were adjusted for height (where appropriate), sex, age, parental social class, and maternal education with interaction terms to assess sex differences.

**Results:**

There was substantial tracking in active travel through adolescence, with 38.5% of males and 32.3% of females consistently walking or cycling to school. In males, a consistently or predominantly active travel pattern was associated with a lower BMI SD score at age 17 (consistently active: adjusted *β*=−0.23; 95% CI −0.40, −0.06; active at two time points: adjusted *β*−0.30; 95% CI −0.50, −0.10) compared to those with a consistently passive pattern. No associations were seen in females.

**Conclusions:**

Maintenance of active travel behaviours throughout adolescence may help to protect against the development of excess BMI in males. In addition to encouraging the adoption of active travel to school, public health messages should aim to prevent drop out from active travel to promote good health in youth.

## Highlights

• Active travel tracks through adolescence.
• Walking tracks more strongly than cycling.
• Active travel is associated with body composition in males.

## 1 Introduction

Young people who are physically active are less likely to be overweight, have an improved body composition and more favourable cardiovascular risk profile than their less active peers (Janssen and LeBlanc, 2010, Ness et al., 2007). However, few young people are sufficiently active to achieve these health benefits. For example, in the UK it is estimated that only 21% of boys and 16% of girls aged 5–15 years achieve recommended daily levels of moderate to vigorous physical activity (MVPA) (Health and Social Care Information Centre, 2012). In addition to generally low levels of physical activity, MVPA declines by approximately 7% per year throughout adolescence (Corder et al., 2013, Dumith et al., 2011).

Walking and cycling to school (active travel) may offer young people an opportunity to incorporate physical activity into their daily lives. Studies utilising objective measures of MVPA have found walking to school to contribute between 24.6 and 40.2% of daily MVPA (Southward et al., 2012, Van Sluijs et al., 2009a). In the UK, studies in primary school-aged children have shown that over 1-year, children who changed from passive travel to walking to school increased MVPA by 12–16% (Cooper et al., 2012, Smith et al., 2011). Furthermore, there is emerging evidence of the health benefits of active travel. In children, some, but not all studies have shown walking and cycling to school to be associated with improved body composition (Andersen et al., 2011, Lubans et al., 2011), while more consistently cycling to school has been shown to be associated with increased cardiorespiratory fitness and a more favourable cardiovascular risk profile (Cooper et al., 2008, Voss and Sandercock, 2010). These findings suggest that active travel to school could be an important factor in the prevention of the decline in MVPA seen through adolescence and may contribute towards improved body composition and cardiovascular health.

However, little is known about the tracking of travel behaviours through adolescence and the long-term impact of maintaining active travel behaviours on health and body composition. The aims of this study therefore are to describe the tracking of travel mode to school throughout adolescence and identify whether maintenance of active travel to school is associated with an improved body composition at age 17.

## 2 Material and methods

### 2.1 Study population

Data presented are from the Avon Longitudinal Study of Parents and Children (ALSPAC), a prospective birth cohort study described in detail elsewhere (Boyd et al., 2013) (http://www.alspac.bris.ac.uk). Briefly, 14,541 pregnant women living in one of the three Bristol-based health districts in the former county of Avon (UK) with an expected delivery date between April 1991 and December 1992 were enroled in the study. During pregnancy and throughout childhood and adolescence, detailed information was collected using self-administered questionnaires, data extraction from medical notes, linkage to routine information systems and at research clinics. Ethical approval for the study was obtained from the ALSPAC Ethics and Law Committee and the Local Research Ethics Committees.

### 2.2 Data collection procedures

The present study is based on responses to questionnaires sent when the participants were aged 12, 14 and 16 years, and on clinical assessments performed when they were aged 17.5 years. Please note that the study website contains details of all the data that are available through a fully searchable data dictionary (http://www.bristol.ac.uk/alspac/) .

### 2.3 Mode of travel

At age 12, the main carer was asked to report the child’s usual travel mode to and from school. The carer indicated, for journeys to and from school separately, whether or not the child used one of six specified modes of transport (walking/cycling/car/public transport/school bus/wheelchair) or another mode (other). Frequency of use was reported in two categories: ‘every or most days’ or ‘some days’. The travel mode with the highest reported frequency was selected as the main mode of transport. If two or more travel modes were reported to occur equally frequently, the travel mode with the lowest energy expenditure per distance travelled was selected in the following order: ‘car’; ‘public transport/school bus/wheelchair/other’; ‘walking’; ‘cycling’ (van Sluijs et al., 2009b). At ages 14 and 16, the participants were asked to report their usual travel mode to and from school, college or work separately and to indicate how they had travelled with the following options: walk all of the way, walk part of the way, public bus, school bus, car/taxi, bicycle, train/metro, skateboard or scooter. If two or more travel modes were selected, the travel mode with the lowest energy expenditure per distance travelled was selected in the following order: ‘car’; ‘public transport/school bus/wheelchair/other’; ‘walking’; ‘cycling’. In ALSPAC, travel mode to school has been shown to correlate highly with travel mode from school, therefore only travel mode to school was used for analyses (van Sluijs et al., 2009b). At each age, travel mode to school was dichotomised into ‘active travel (walking or cycling)’ and ‘passive travel (all other options)’. Due to difficulties in assigning public transport to active or passive travel, children who reported this travel mode were not included in our analyses. Subsequently adolescents were classified into one of the four groups to reflect the pattern of travel to school over time: used active modes at all three time points (consistently active), used passive modes at all three time points (consistently passive), were active at two time points or active at one time point.

### 2.4 Body composition

Body composition was measured at the 17-year old clinics. Weight was measured using a Tanita TBF 305 body fat analyser and weighing scales (Tanita, UK). Height was measured with shoes and socks removed using a Harpenden Stadiometer (Holtain, UK). BMI was calculated as weight (in kilograms) divided by height (in metres) squared. Fat mass was measured using a Lunar Prodigy DXA scanner (GE Medical Systems, http://www.hehealthcare.com).

### 2.5 Covariates

In the 32-week antenatal questionnaire, the mother was asked to record the actual occupation of both herself and her partner, which were used to allocate them into three social-class groups (classes I/II, classes III manual and non-manual and classes IV/V) using the 1991 UK census classification. The mother was also asked to record her highest educational level (none/Certificate of Secondary Education (CSE)/Ordinary (O) level (national school exams at age 16)/vocational/Advanced (A) level (national school exams at age 18)/university degree).

### 2.6 Statistical analyses

The data were summarised using means and standard deviations (SD) for continuous variables, medians and inter-quartile ranges for skewed variables (fat mass at age 17 years only) and proportions for categorical variables. Generalised estimating equations (GEE) were used to calculate longitudinal tracking coefficients (expressed as odds ratios and 95% confidence intervals and interpretable as coefficients of stability over time), giving the association between travel mode to school at age 12 with travel mode to school at all other ages (Twisk et al., 1996, Zeger et al., 1985). This gives the odds of a subject who uses a particular travel mode at age 12 maintaining that behaviour across the three time points relative to the odds of a subject who did not use that travel mode at age 12. Models were adjusted for actual age at measurement. Internally derived SD (Z) scores were calculated for BMI, and fat mass to allow for comparison of the regression coefficients across outcome measures (Leary et al., 2006). Logged fat mass was used to calculate the SD score due to the skewness of the distribution. The associations between longitudinal travel patterns to school and BMI and fat mass were assessed using linear regression analysis. Interaction terms were included to test the association between pattern of active travel and body composition for an interaction with gender. There was evidence of an interaction (*p*<0.1) for BMI and therefore all analyses were performed in males and females separately. Models were initially adjusted for height (fat mass) and age at clinic attendance, then additionally for social factors (social class and maternal education). All analyses were conducted using STATA 12 (College Station, TX; StataCorp) in 2014.

## 3 Results

### 3.1 Demographics

Five thousand and eighty-one singletons attended the 17-year follow-up clinic. Of those attending, estimates of body composition from the DXA scan were available for 4,848 (95.3%). A total of 2,670 (55.1%) of these provided complete data on travel mode to school. Of these, 644 reported using public transport or other modes of transport and were not included in the analyses. The final sample for analysis was 2,026 participants, and descriptive characteristics of these participants are shown in Table 1. A higher proportion of the participants who provided complete data and were included in the analyses were girls and were from a higher social class compared to participants not included in the analyses (data not shown).

### 3.2 Mode of travel

The proportion of children participating in each travel behaviour at ages 12, 14 and 16 is displayed in Table 2. At age twelve, 48.9% of males and 48.5% of females reported walking to school, with the proportion decreasing to 38.7% and 41.4% at age 16. The most common travel pattern reported through adolescence for males was consistently active (38.5%) and consistently passive in females (37.1%). The tracking coefficients for overall and individual travel behaviours and patterns of travel through adolescence are displayed in Table 3. Tracking coefficients can be interpreted as coefficients of stability over time and give us the odds of a subject who participates in a particular travel behaviour at age 12 maintaining that behaviour across the 3 time points relative to an individual who did not participate in that particular behaviour at age 12. The overall tracking coefficient for maintenance of active travel through adolescence was an odds ratio of 1.07 (95% CI 1.05, 1.10) for males and 1.07 (95% CI 1.05, 1.10) for females in those who were active travellers at age 12 compared to those who were not. The strength of the tracking coefficient varied between individual travel behaviours. The strongest positive tracking appeared to be for walking with an odds ratio of maintaining walking behaviour throughout the 4 year period of 1.09 (95% CI 1.09, 1.13) in males and 1.07 (95% CI: 1.04, 1.09) in females, for those who walked at age 12 compared to those who did not.

### 3.3 Association between travel pattern and body composition

The associations between travel pattern through adolescence and body composition at age 17 are shown in Table 4. In males, choosing either consistently active modes of transport, or active modes of transport at two ages through adolescence was associated with a reduced BMI SD score at age 17 (adjusted β-0.23; 95% CI −0.40, −0.06 for consistently active and adjusted *β* −0.30; 95% CI −0.50, −0.10 for active at two time points) compared to those who were consistently passive. No associations between travel pattern and body composition variables were observed in females.

## 4 Discussion and conclusion

This study investigated the tracking of active travel through adolescence and its association with body composition at age 17 in a large UK birth cohort. Few prior studies have described the maintenance of travel behaviours through adolescence. We found that active travel showed substantial tracking, with 38.5% of males and 32.3% of females choosing active modes of travel at every age and the odds of maintaining active travel were increased for those who chose active modes of travel at age 12. These findings are similar to those in a recent study in Brazilian adolescents, which found substantial evidence of tracking of active travel from ages 11 to 18 years (Martinez-Gomez et al., 2014). Walking demonstrated the most substantial positive tracking of all travel behaviours, while weak tracking coefficients for passive travel behaviours such as car travel were observed. Males who chose either consistently or predominantly (at least twice) active modes of transport through adolescence had a reduced BMI SD score at age 17 compared to males who chose consistently passive travel modes. There were no associations between maintenance of active travel and body composition in females. These findings suggest that once established, active travel behaviours are maintained throughout adolescence and may help to protect against the development of excess BMI in males.

There is a consistent literature on the benefits of active commuting for improved physical activity, with active travellers accumulating more daily MVPA than those using motorised transport in the majority of studies. However, associations with health outcomes are less clear. Approximately fifty percent of cross-sectional studies report significant associations between active travel to school and weight status or body composition, but studies are often limited by small numbers and poor study design (Lubans et al., 2011). A small number of longitudinal studies have also investigated the association between active travel to school and health outcomes. Adoption or maintenance of cycling to school by European adolescents has been shown to be associated with improved cardiorespiratory fitness (Chillón et al., 2012, Cooper et al., 2008) and cardiovascular risk profiles (Andersen et al., 2011). A longitudinal study in 6–8 year old children in Canada found that sustained active travel was associated with a more healthy BMI trajectory across the early school years (Pabayo et al., 2010), whilst in Brazil, both cross-sectional and prospective associations between active travel (walking/cycling) and central body fat were observed in males only (Martinez-Gomez et al., 2014). Similarly, in the current study the association between active travel and BMI SDS was observed in the males only. However, no differences in fat mass were seen. Having a lower BMI with the same fat mass is not necessarily healthier and therefore these results should be interpreted with a degree of caution.

The factors underlying the sex differences seen in ours and the Brazilian study are unclear, with Martinez-Gomez et al. suggesting that males may participate in more intense active travel, contributing more to their daily requirement of moderate to vigorous physical activity (MVPA). However, previous research exploring differences in MVPA accumulated during walking to school found no gender differences in the intensity of physical activity during the journey in a sample of 11–12 year old British schoolchildren (Southward et al., 2012). Alternatively, the lack of association with BMI observed in girls may be as a result of compensation in physical activity. It has previously been suggested that children and adolescents may compensate for time spent in active travel by being less active during school times or in the after-school period, with the possibility of a gender difference in the level of compensation (Panter et al., 2011). However, studies utilising objective measures of the journey to school and MVPA have failed to show evidence of this compensation, instead demonstrating that children of both genders who engaged in active travel accrue more MVPA over the course of the day compared to those who don’t (Southward et al., 2012). Notably, since overall daily MVPA was lower for the girls, active travel made a greater contribution to MVPA in girls than in boys, and the contribution of active travel to daily MVPA may increase through adolescence as MVPA declines and gender differences in MVPA become more apparent (Dumith et al., 2011).

This study is the first to assess longitudinal associations between travel patterns through adolescence and body composition outcomes in a UK cohort. The large sample size, prospective nature and comprehensive, objectively measured outcomes contribute to the strength of the study. However, this study also has some limitations. Travel mode to school was recorded using a self-report measure which changed from parent report at age 12 to adolescent report at ages 14 and 16. Although we are unable to examine the convergent validity of this measure within the current study, previous research has demonstrated good agreement between parent and child reports of travel mode to and from school (Evenson et al., 2008). Due to low numbers utilising certain travel modes, travel mode to school was dichotomised and therefore limits any conclusions drawn about the effects of mixed methods of travel or within different types of active or passive travel (i.e. walking versus cycling). Furthermore we elected to remove from the analysis children who utilised public transport which is likely to have a combination of active and passive components. Future research should aim to establish the contribution of mixed methods of travel and public transport to daily MVPA. Low levels of cycling in UK schoolchildren limit the ability to examine the separate effects of walking and cycling on body composition; however, evidence from European countries such as the Denmark suggests that the higher intensity of cycling will have a greater, beneficial effect on cardiorespiratory fitness and body composition (Cooper et al., 2006, Cooper et al., 2008).

Distance to school has been shown to be a key determinant of both the uptake and maintenance of active travel to school (Panter et al., 2011, Van Sluijs et al., 2009a), and will also impact upon the contribution of active travel to daily MVPA (Cooper et al., 2012). The relationship between maintenance of active travel through adolescence and BMI at age 17 is likely to be affected by the dose of active travel performed which in turn is influenced by both the distance of the journey and intensity of the activity performed. The current study is limited by a lack of objective measurement of distance to school and MVPA. However, the primary aim of the study was to demonstrate whether maintenance of active travel through adolescence was associated with body composition. The results suggest that in boys maintenance of active travel through adolescence may be beneficial for BMI; however, further work using objective measurements of MVPA and distance to school is needed to identify the dose of active travel and MVPA required for these beneficial associations to be seen.

The current sample contains a low proportion of ethnic minority children and therefore findings may not be generalisable to the rest of the UK and around the world. Furthermore, we were unable to collect complete travel mode and body composition data on a substantial number of children initially enroled in the ALSPAC cohort and therefore the study may be affected by selection bias. In addition, while we were able to collect data on socioeconomic measures such as maternal education, and IMD, there may be some residual confounding from additional factors such as the built environment or council provision of transport to school, factors which may affect both the decision to uptake and maintain active travel. In Bristol, travel support is provided by the city council for all children living further than 3 miles from school and for children from low-income families who live further than 2 miles from school (www.bristol.gov.uk/page/children-and-young-people/school-travel).

There is growing evidence of the potential for active travel in childhood and adolescence increasing MVPA, improving cardiorespiratory fitness and improving body composition. Despite this, interventions to promote active travel to school are few in number and limited by small size and poor study design (Chillón et al., 2011, Larouche et al., 2014). Systematic reviews have identified intervention studies primarily conducted in primary-school aged children, with an observed effect size ranging from 3% to 65% (Chillón et al., 2011, Larouche et al., 2014) improvement. Further studies have identified a number of social and environmental predictors of active travel including distance to school, and parental perceptions of convenience and safety (Panter et al., 2013, Van Dyck et al., 2010). Therefore, for more effective promotion of the uptake and maintenance of active travel, high-quality interventions which take into account the potential correlates of active travel are required.

In conclusion, travel mode to school demonstrates tracking through adolescence and maintenance of active travel through adolescence is associated with a reduced BMI SD score at age 17 in males. Although no effects were seen in females, a growing evidence base suggests that interventions to promote the uptake and maintenance of active travel through the adolescent period may offer protection against the development of excess BMI. The high tracking of travel mode through adolescence suggests that early intervention during the transition from primary to secondary school followed by promotion of maintenance of active travel through adolescence may be of greatest benefit.

## Conflict of interest

The authors declare that there is no conflict of interest associated with this manuscript. The work of all authors was supported by National Institute for Health Research (NIHR) Bristol Nutrition Biomedical Research Unit based at University Hospitals Bristol NHS Foundation Trust and the University of Bristol. The views expressed are those of the author(s) and not necessarily those of the NHS, the NIHR or the Department of Health. The study sponsor had no role in the study design; collection, analysis, and interpretation of data; writing the paper; and the decision to submit the paper for publication.

## Financial disclosure

No financial disclosures were reported by the authors of this paper.

## Figures and Tables

**Table 1:** Descriptive characteristics of ALSPAC sample included in analyses (Bristol, UK 2008–2011).

	**Total sample**	**Males**	**Females**	***P* value**^a^
	**Mean (SD)**	**Mean (SD)**	**Mean (SD)**	
N (%)	2,026	838 (41.3)	1,188 (59.858.6)	
Age (yrs)	17.7 (0.4)	17.7 (0.4)	17.7 (0.4)	0.170
Height (m)	1.71 (0.1)	1.79 (0.1)	1.66 (0.1)	<0.01
Weight (kg)	66.0 (13.2)	71.8 (12.7)	61.9 (11.9)	<0.01
**Social Class in pregnancy (%)**				
I//II	47.1	49.8	45.2	0.011
III manual and non-manual	45.3	44.3	46.0	
IV/V	7.6	5.9	8.8	
**Maternal Education (%)**				
None/CSE/O level (pre 16)	40	38.8	40.9	0.533
Vocational	5.5	5.4	5.6	
A levels (post 16)	30.4	30.4	29.5	
Degree (University)	24.1	24.0	24.0	
**Body composition**				
Body mass index (BMI) (kg/m^2^)	22.5 (3.9)	22.4 (3.6)	22.6 (4.1)	0.13
Fat mass (kg)^b^	16.2 (10.8–23.0)	10.6 (6.8–16.8)	19.3 (14.7–25.0)	<0.01

a From *t*-tests for continuous variables, chi-squared test for differences for categorical variables and Wilcoxon rank sum for skewed variables.

b Median and interquartile ranges are displayed for skewed variable fat mass.

**Table 2:** Travel mode to school throughout adolescence and travel pattern through adolescence in ALSPAC sample (Bristol, UK, 2008–2011).

**Travel mode**	**Total sample (%)**	**Males (%)**	**Females (%)**	***P* value**^a^
**12 years**				
Walk	48.7	48.9	48.5	<0.01
Cycle	4.2	7.3	2.0	
Car	32.5	30.6	33.9	
School bus	14.6	13.3	15.6	
**14 years**				
Walk	50.8	50.8	50.8	<0.01
Cycle	4.4	8.5	1.5	
Car	25.9	24.1	27.1	
School bus	18.9	16.6	20.5	
**16 years**				
Walk	40.3	38.7	41.4	<0.01
Cycle	4.8	10.1	1.1	
Car	37.1	35.8	38.0	
School bus	17.8	19.5	15.4	
**Travel pattern through adolescence**				
Consistently active^b^	34.9	38.5	32.3	<0.01
Active at 2 time points	18.3	18.9	17.9	
Active at 1 time point	12.0	11.0	12.7	
Consistently passive	34.9	31.6	37.1	

a From chi-squared test for differences.

b defined as walking or cycling.

**Table 3:** Mode of travel to school at age 12 and tracking coefficient for travel mode through adolescence (Bristol, UK, 2008–2011).

**N=2,026**	**Participation at age 12**		**Odds of tracking**^a^^**,**^^b^	
**Travel to school**	**N**	**%**	**OR (95% CI)**	**P value**
*Active Travel*				
Males	471	56.2	1.07 (1.05, 1.10)	<0.001
Females	600	72.5	1.07 (1.05, 1.10)	<0.001
*Walking*				
Males	410	48.9	1.09 (1.10, 1.13)	<0.001
Females	576	48.5	1.07 (1.04, 1.09)	<0.001
*Cycling*				
Males	61	7.3	0.93 (0.88, 0.99)	0.020
Females	24	2.0	1.14 (1.00, 1.28)	0.049
*Car*				
Males	256	30.6	0.94 (0.91, 0.97)	<0.001
Females	403	33.9	0.95 (0.93, 0.98)	<0.001
*School Bus*				
Males	111	13.3	0.97 (0.93, 1.00)	0.127
Females	185	15.6	0.95 (0.92, 0.98)	0.002

a Adjusted for exact age at measurement.

b Tracking coefficients for maintenance of travel behaviours calculated with generalised estimating equations over a 4-year period from age 12 to age 16 years.

**Table 4:** The association between longitudinal patterns of active travel to school and body composition SD scores (Bristol, UK, 2008–2011).

**Group (n)**	**Model**		**BMI SD**		**Fat mass SD**	
			**β (95% CI)**	**p**	**β (95% CI)**	**p**
Males (1,052)	Partially adjusted^a^	**Ref:** Consistent passive				
		Consistent active	−0.18 (−0.35, −0.02)	0.026	−0.05 (−0.21, 0.12)	0.583
		Active at 2 time points	−0.21 (−0.41, −0.02)	0.032	−0.02 (−0.20, 0.18)	0.858
		Active at 1 time point	0.04 (−0.19, 0.28)	0.719	0.19 (−0.05, 0.43)	0.117
	Fully adjusted^b^	**Ref:** Consistent passive				
		Consistent active	−0.23 (−0.40, −0.06)	0.007	−0.10 (−0.27, 0.07)	0.257
		Active at 2 time points	−0.30 (−0.50, −0.10)	0.004	−0.09 (−0.30, 0.11)	0.364
		Active at 1 time point	−0.02 (−0.26, 0.22)	0.884	0.14 (−0.11, 0.38)	0.266
Females (1,476)	Partially adjusted^a^	**Ref:** Consistent passive				
		Consistent active	0.04 (−0.10, 0.18)	0.568	0.01 (−0.15, 0.12)	0.847
		Active at 2 time points	0.14 (−0.03, 0.30)	0.100	0.08 (−0.09, 0.24)	0.354
		Active at 1 time point	0.04 (−0.14, 0.22)	0.668	−0.001 (−0.19, 0.18)	0.991
	Fully adjusted^b^	**Ref:** Consistent passive				
		Consistent active	0.07 (−0.07, 0.21)	0.348	0.00 (−0.14, 0.14)	0.999
		Active at 2 time points	0.12 (−0.05, 0.29)	0.233	0.06 (−0.11, 0.22)	0.513
		Active at 1 time point	0.05 (−0.14, 0.24)	0.617	0.00 (−0.19, 0.19)	0.999

a Adjusted for age at time of 17 year clinic, and height.

b Additionally adjusted for social class, and maternal education. NB. BMI, and fat mass are all expressed as internally derived SD scores, allowing for comparison across outcome measure.
